# Supplementary material for: Identification of Spatial Specific Lipid Metabolic Signatures in Long-Standing Diabetic Kidney Disease
Source: Metabolites. 2024 Nov 20;14(11):641. doi: 10.3390/metabo14110641 (PMC11596753; doi:10.3390/metabo14110641)
Supplement: Supplementary file 1 [file metabolites-14-00641-s001.zip › Supplemental Figures.pdf]

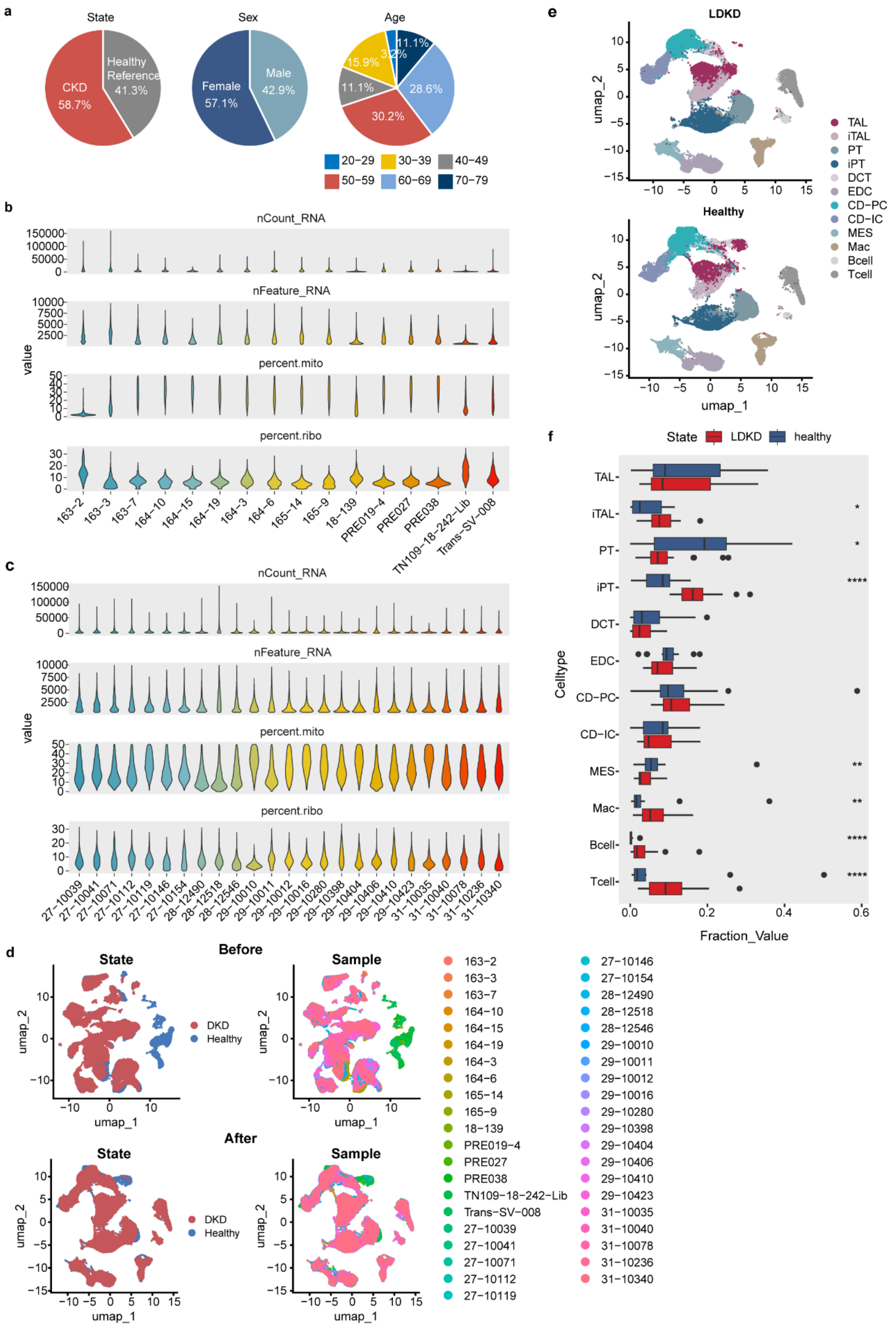

Supplementary Figure 1. Single-cell RNA Sequencing (scRNA-seq) analysis reveals kidney cell types in LDKD and healthy donors. (a) Clinical profiles of participants in the study. (b-c) Violin plot of UMI counts, gene numbers, mitochondrial gene proportions, and ribosomal gene proportions in samples from healthy (b) and LDKD participants. (d) UMAP plot of LDKD and healthy samples before (upper) and after (lower) batch effect correction. (e) UMAP plot of kidney cells in LDKD and healthy samples respectively. Different colors correspond to distinct cell types. (f) The proportions of cell types in LDKD and health samples. Wilcoxon test. Only significant p-values are displayed on the right side of each cluster.

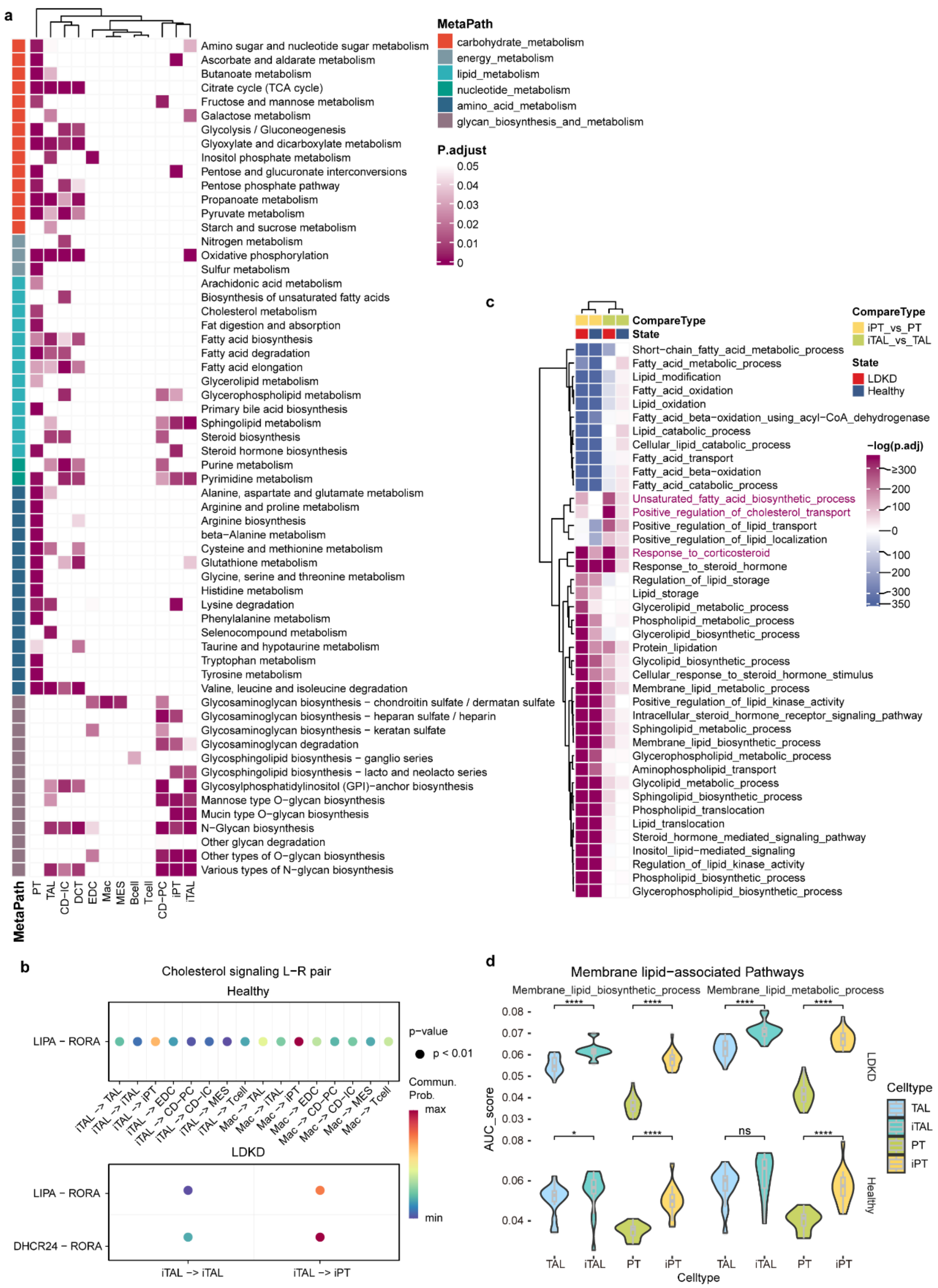

Supplementary Figure 2. Metabolic profiles and pathway activities of kidney injury-associated cell types in LDKD and healthy samples. (a) Heatmap showing the KEGG enrichment analysis results of metabolic pathways across distinct cell types. Color intensity represents pathway significance, with darker shades indicating higher significance. (b) Significant cholesterol metabolism ligand-receptor pairs in LDKD and healthy samples. Permutation test. (c) Pathway activity comparison between iTAL vs. TAL and iPT vs. PT in LDKD and healthy samples. AUC-based activity changes shown in dark pink (positive) and dark blue (negative), with intensity reflecting adjusted p-values. Only pathways exceeding the mean -log10 (p.adj) value in any comparison are shown. (d) Violin plot of membrane lipid pathway activities in LDKD (upper) and healthy (lower) samples across iTAL vs. TAL and iPT vs. PT. Wilcoxon test.

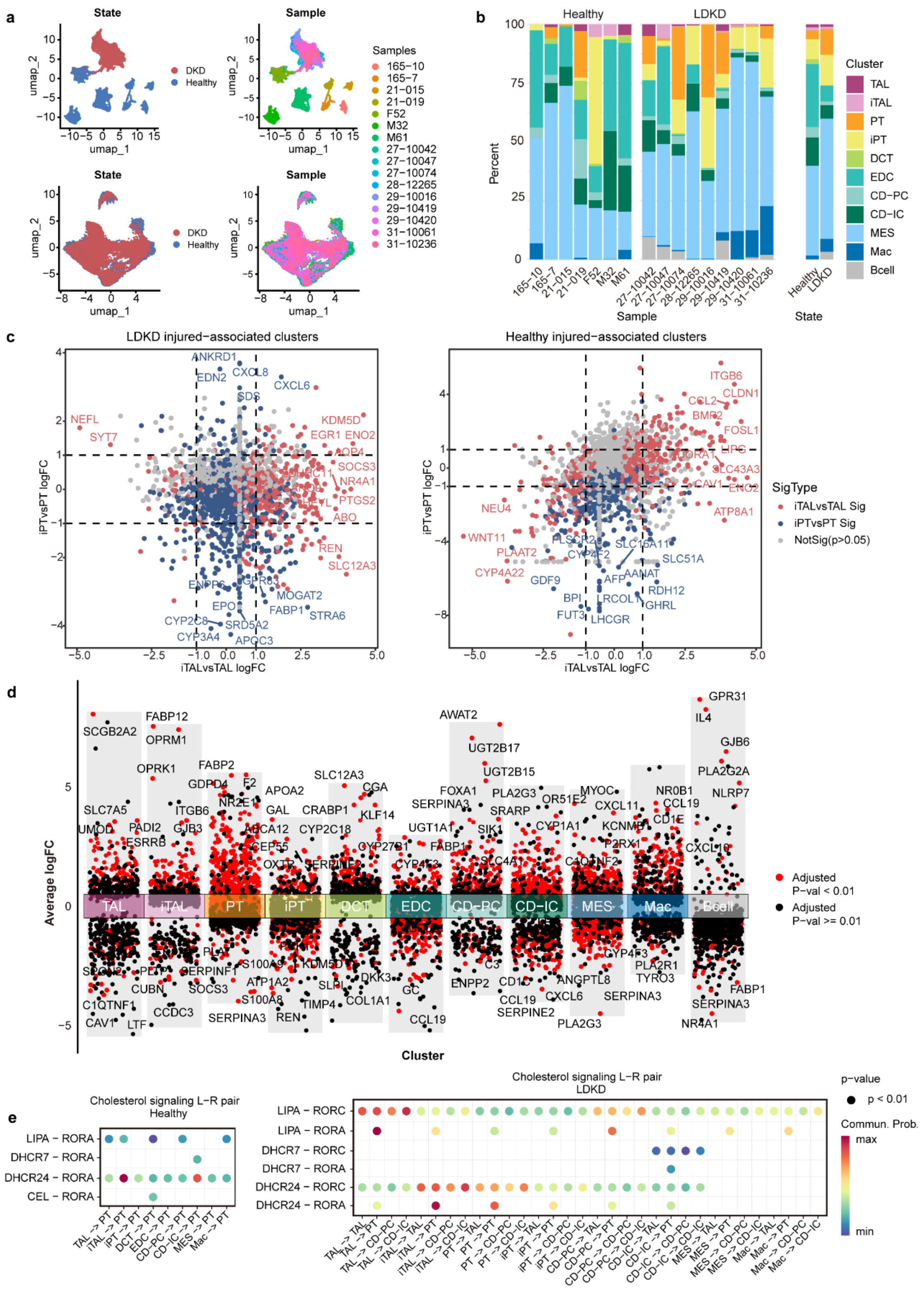

Supplementary Figure 3. Spatial transcriptomics analysis for LDKD and healthy samples. (a) UMAP plot of LDKD and healthy samples before (top) and after (bottom) batch correction. (b) Bar plot of the composition of different cell types in each sample. Only cells with a final proportion exceeding 50% are included. (c) Scatter plot of differentially expressed genes (DEGs) in two comparisons between LDKD and healthy samples. The x-axis represents the avg_logFC for iTAL vs TAL, and the y-axis for iPT vs PT. Point colors indicate significance type, and genes are from lipid-associated pathways. P-values in both comparisons are based on the Wilcoxon test. (d) Highlight of significantly altered genes per cell type. Different colors denote distinct significance types (Wilcoxon test, threshold = 0.5). Top 5 highest/lowest fold change genes are labeled for each cell type. (e) Significant ligand-receptor interactions in the cholesterol metabolism pathway network for LDKD and healthy samples. Significant pairs are identified via permutation testing.

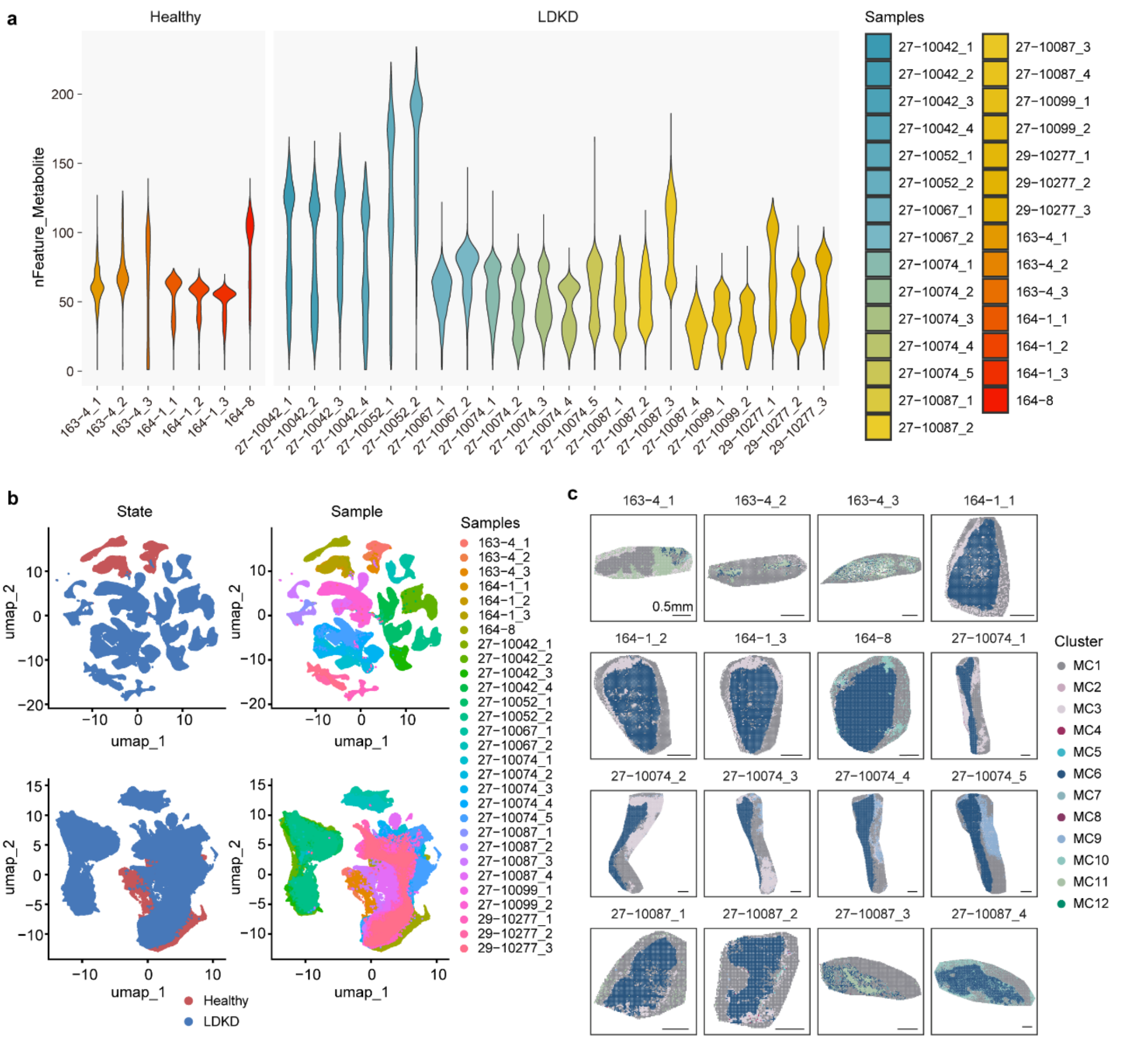

Supplementary Figure 4. Spatial metabolomics analysis for LDKD and healthy samples. (a) Violin plot of metabolite feature numbers in spatial metabolomics samples from LDKD patients and healthy donors. (b) UMAP plot of LDKD and healthy samples before (upper) and after (lower) batch effect correction. (c) Spatial distribution of MCs in samples from remaining LDKD patients and healthy donors. Scale bar, 0.5 mm.

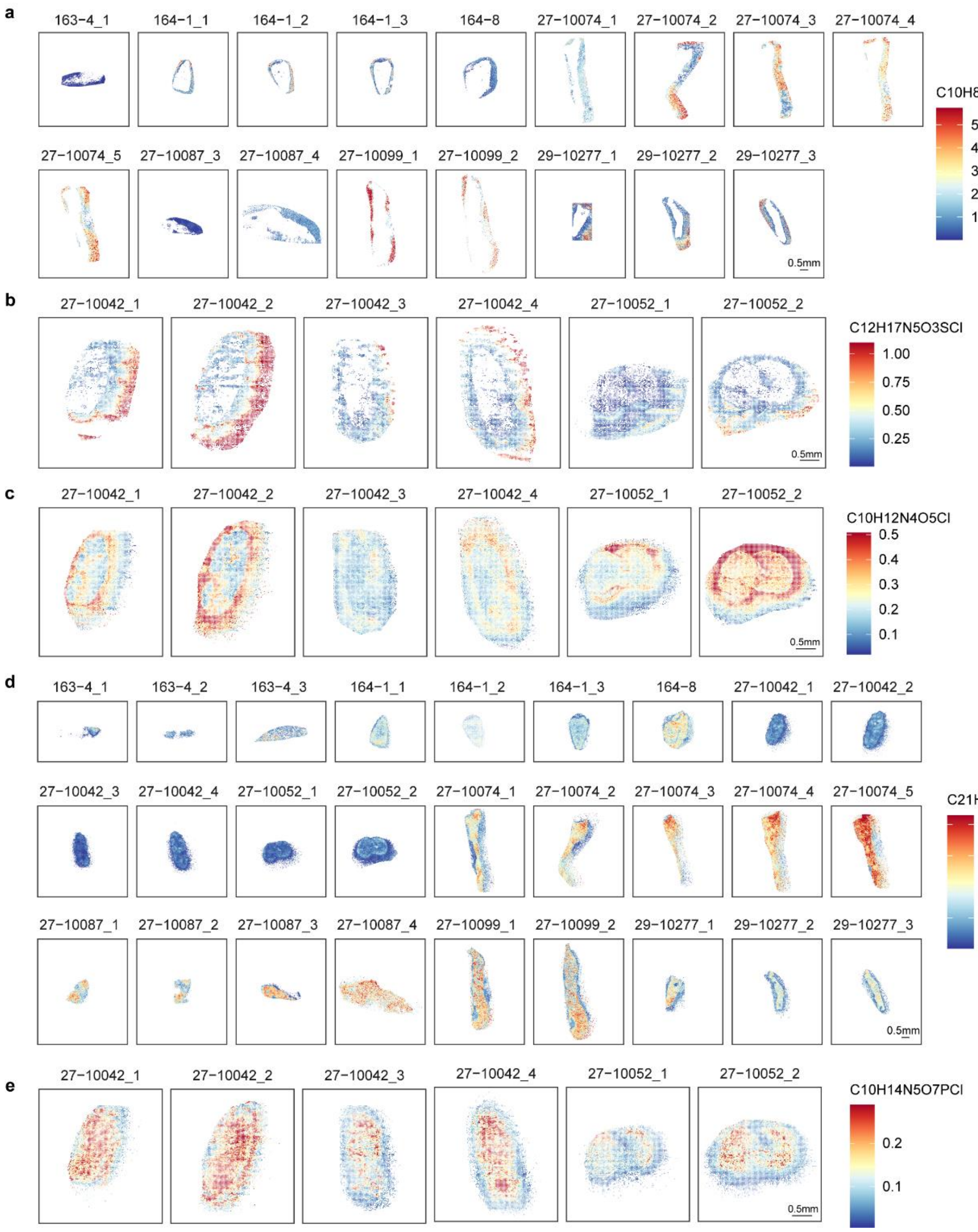

Supplementary Figure 5. Spatial distribution of characteristic metabolites for distinct MCs. Spatial distribution of the metabolites: (a) $C_{10}H_8NO_4$, a characteristic metabolite for MC1; (b) $C_{12}H_{17}N_5O_3SCl$, a characteristic metabolite for MC2; (c) $C_{10}H_{12}N_4O_5Cl$, a characteristic metabolite for MC4; (d) $C_{21}H_{38}O_6P$, a characteristic metabolite for MC6; (e) $C_{10}H_{14}N_5O_7PCl$, a characteristic metabolite for MC7. Scale bar, 0.5 mm.

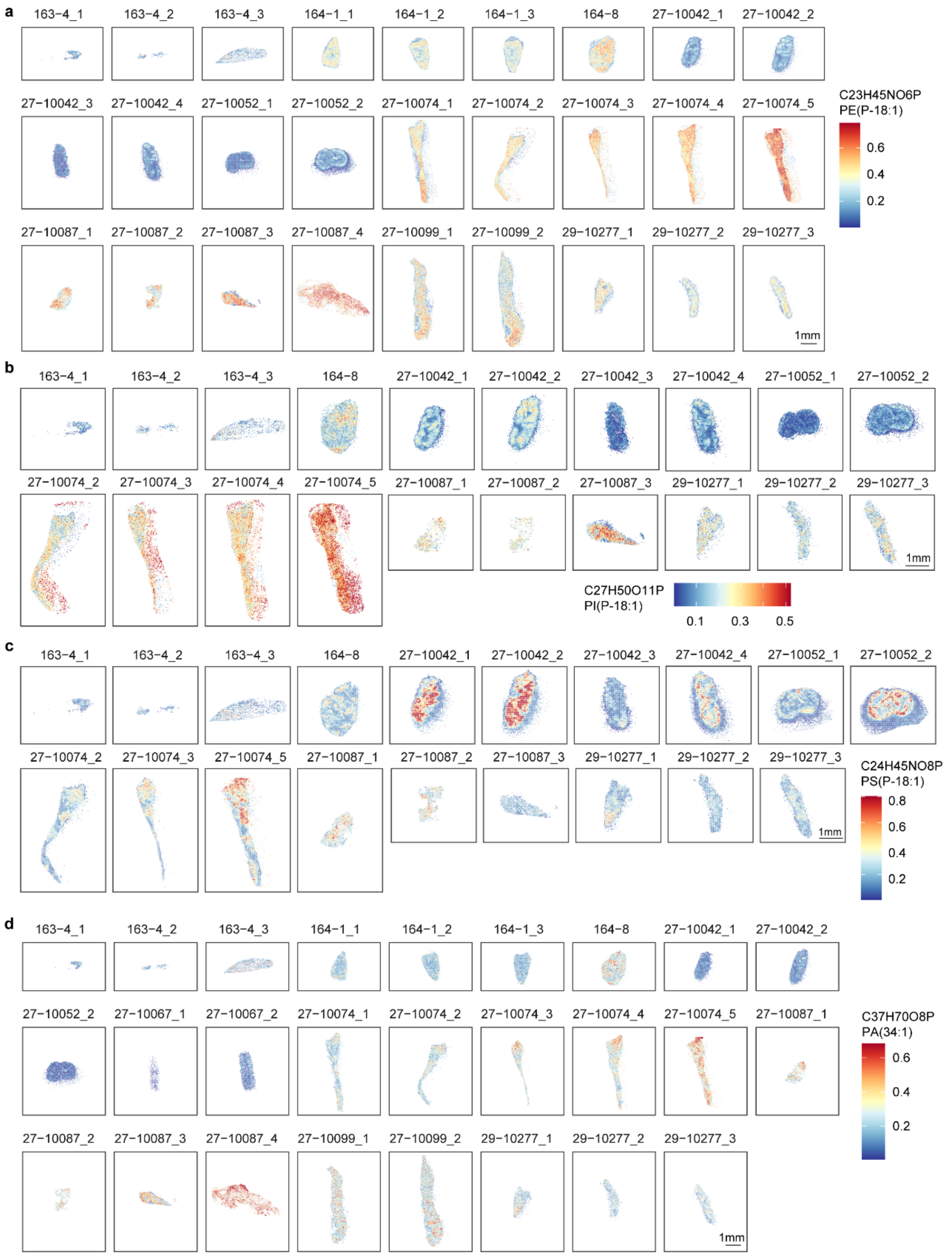

Supplementary Figure 6. Spatial distribution of characteristic metabolites for lipid classes. Spatial distribution of the metabolites: (a) $C_{23}H_{45}NO_6P$, annotated as PE(P-18:1); (b) $C_{27}H_{50}O_{11}P$, annotated as PI(P-18:1); (c) $C_{24}H_{45}NO_8P$, annotated as PS(P-18:1); (d) $C_{37}H_{70}O_8P$, annotated as PA(34:1). Scale bar, 1 mm.

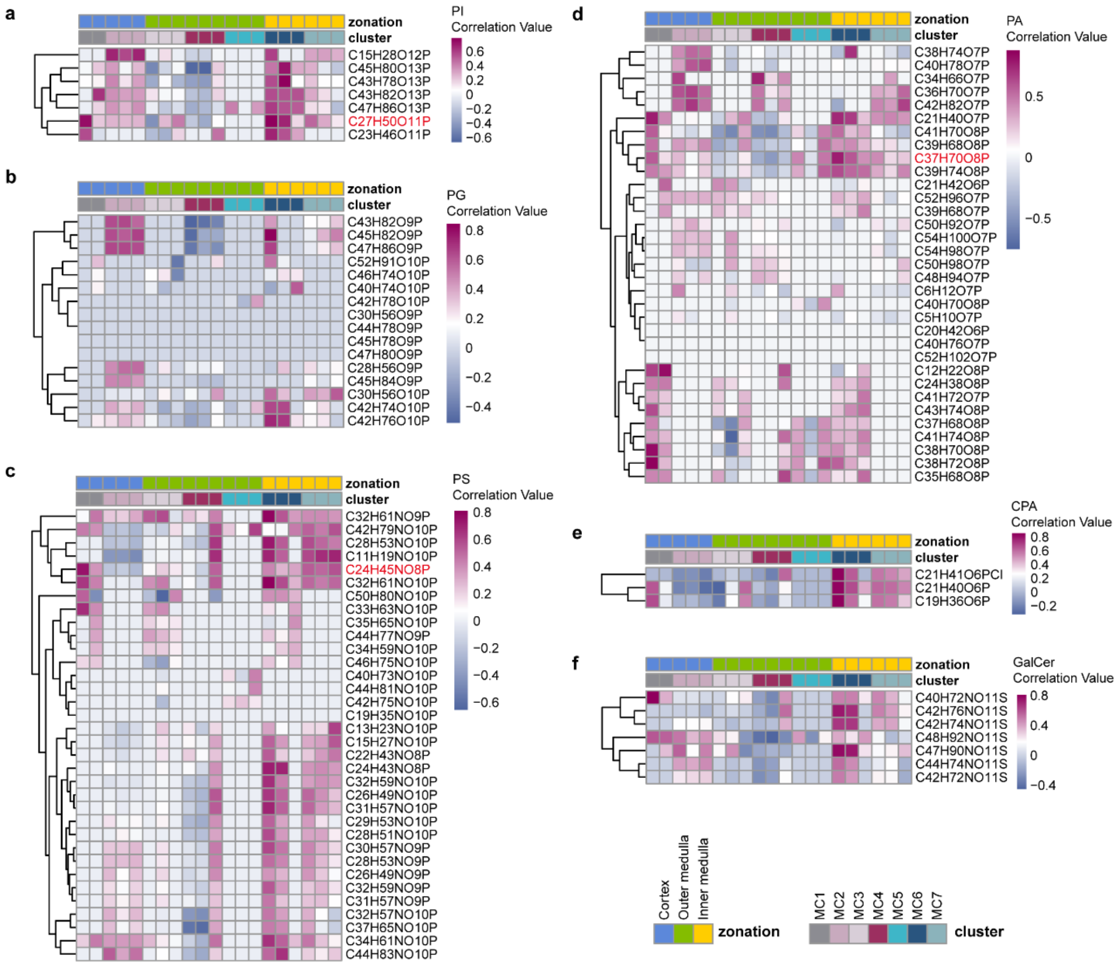

Supplementary Figure 7. Spatial distribution similarities between lipids and characteristic metabolites of different MCs in LDKD. Spatial distribution similarity of PI (a), PG (b), PS (c), PA (d), CPA (e), and GalCer (f) with metabolic clusters in LDKD. The heatmap colors represent the correlation between each metabolite and different metabolic clusters, defined by their spatial distribution similarity. Kidney regions and metabolic clusters are distinguished by distinct colors and annotations. PI, phosphatidylinositol; PS, phosphatidylserine; PG, phosphatidylglycerol; PA, phosphatidic acid; CPA, cyclic phosphatidic acid; GalCer, galactosylceramide. For each MC, 2 or 3 characteristic metabolites were selected to characterize the spatial distribution. The characterized metabolites shown in FigS6 are highlighted in red.
